# Infants’ Food

**Published:** 1858-04

**Authors:** 


### INFANTS' FOOD.

WHEN it is necessary to feed infants artificially, and cows' milk is used, it should be first boiled, then skimmed, then sweetened a little with sugar, and next a little salt added, not enough to give it a saltish taste; milk thus prepared will not only prevent the indigestion and consequent acidity, flatulence, colic, diarrhea, &c., from which sucking children suffer so much, but will actually cure them.

A hearty infant will swallow, during the first year of its life, fourteen hundred pounds of milk, in which are twenty-one pounds of cheese, thirty pounds of butter, and a hundred and twelve pounds of sugar. At six cents a quart, with the necessary sweetening, each "*dear*" little creature costs, for food alone, fifty dollars for the first year.

